# Simulative Evaluation of a Joint-Cartesian Hybrid Motion Mapping for Robot Hands Based on Spatial In-Hand Information

**DOI:** 10.3389/frobt.2022.878364

**Published:** 2022-06-22

**Authors:** Roberto Meattini, Davide Chiaravalli, Gianluca Palli, Claudio Melchiorri

**Affiliations:** DEI—Department of Electrical, Electronic and Information Engineering “Guglielmo Marconi”, University of Bologna, Bologna, Italy

**Keywords:** multifingered hands, human-centered robotics, grasping, dexterous manipulation, telerobotics and teleoperation

## Abstract

Two sub-problems are typically identified for the replication of human finger motions on artificial hands: the measurement of the motions on the human side and the mapping method of human hand movements (primary hand) on the robotic hand (target hand). In this study, we focus on the second sub-problem. During human to robot hand mapping, ensuring natural motions and predictability for the operator is a difficult task, since it requires the preservation of the Cartesian position of the fingertips and the finger shapes given by the joint values. Several approaches have been presented to deal with this problem, which is still unresolved in general. In this work, we exploit the spatial information available in-hand, in particular, related to the thumb-finger relative position, for combining joint and Cartesian mappings. In this way, it is possible to perform a large range of both volar grasps (where the preservation of finger shapes is more important) and precision grips (where the preservation of fingertip positions is more important) during primary-to-target hand mappings, even if kinematic dissimilarities are present. We therefore report on two specific realizations of this approach: a distance-based hybrid mapping, in which the transition between joint and Cartesian mapping is driven by the approaching of the fingers to the current thumb fingertip position, and a workspace-based hybrid mapping, in which the joint–Cartesian transition is defined on the areas of the workspace in which thumb and fingertips can get in contact. The general mapping approach is presented, and the two realizations are tested. In order to report the results of an evaluation of the proposed mappings for multiple robotic hand kinematic structures (both industrial grippers and anthropomorphic hands, with a variable number of fingers), a simulative evaluation was performed.

## 1 Introduction

The mapping of finger motions on multi-articulated robot hands is nowadays an open problem in the robotics community (Shahbazi et al., 2018; Carfì et al., 2021). Indeed, a general mapping solution for the replication of human primary hand (PH) motions onto a robotic target hand (TH), ensuring ease of use and good motion reproducibility, is a very challenging target to be achieved. The presence, in real situations, of unavoidable kinematic dissimilarities between PH and TH does not allow to precisely replicate PH finger motions onto TH fingers with a direct identity mapping, and some adaptation and/or interpretation must be adopted (Gioioso et al., 2012). Therefore, despite several advances during the last years, it results from evidence that developing TH to RH mapping strategies is a non-trivial and both conceptual and analytical problem (Meeker et al., 2018).

The two principal fields in which human-to-robot hand mapping methods used are teleoperation and learning by demonstration (Dillmann et al., 2000). In teleoperation applications, data measured from the operator’s human hand are used to control in real-time motion of a robot hand. Differently, in learning by demonstration applications, motion measurements from the human hand are exploited as a source of human skill information to improve the dexterity and the behavior of autonomous robot hands. In the literature, several works have attempted to design mapping solutions such that to obtain correct and predictable behaviors on the TH. Two fundamental sub-problems have been considered for human-to-robot hand mapping: 1*)* the measurement of human PH motions using appropriate kinematic modeling and sensor equipment and 2*)* the design of a mapping algorithm to obtain a proper imitation of PH motions on the robot TH. In this study, we will not focus on the former sub-problem, which has been extensively addressed using sensorized gloves (Cobos et al., 2010; Geng et al., 2011; Bianchi et al., 2013), vision systems (Gupta and Ma, 2001; Infantino et al., 2005; Wachs et al., 2005), hand exoskeletons (Bergamasco et al., 2007), and advanced calibration procedures (Rohling and Hollerbach, 1993). We will instead focus on the second sub-problem, for the reason that it is still lacking a general solution (Colasanto et al., 2012).

Among the many approaches that have been presented in the literature for PH to TH motion mapping, the simplest realization is the direct joint mapping (Speeter, 1992), in which joint angles of the PH are directly imposed onto the robot TH. Direct joint mapping presents the advantage of being rapidly implementable, while preserving PH finger shapes on anthropomorphic TH, allowing better reproduction of gestures and power grasps (Cerulo et al., 2017). Additionally, it generally permits an increased predictability of TH behaviors, allowing the human operator to more easily compensate for inaccurate behaviors by moving ones hand (Speeter, 1992). The second simplest mapping approach is the direct Cartesian mapping (Rohling and Hollerbach, 1993), which consists in computing Cartesian positions and orientations of the PH fingertips (i.e., the fingertip *poses*) by means of forward kinematics, and directly imposing them on the TH fingertips, followed by the computation of the TH joint angles via inverse kinematics. Direct Cartesian mapping is more appropriate for the execution of precision grasps and in-hand manipulation since it preserves the PH fingertip positions on the TH. However, on the other hand, it does not guarantee, in general, the preservation of the PH finger shapes, making difficult the correct execution of power grasps, non-prehensile manipulation, and gestures (Chattaraj et al., 2014). More advanced mapping approaches are based on attenuating the problem of PH-TH kinematic dissimilarities by using a suitable task-oriented description of the finger motions. This is the case of the object-based mapping method (Gioioso et al., 2013), in which PH finger motion information is encapsulated within a virtual object defined in the PH workspace, which is then reported in the TH workspace in order to impose coordinated finger motions. This mapping presents the advantage of being implementable for non-anthropomorphic TH, in which case direct joint and direct Cartesian mappings are particularly unsatisfactory. However, when the precision of single fingertips is required, or purposes different from grasping objects are desired, the object-oriented mapping can produce highly unintuitive behaviors of the TH (Meeker et al., 2018) critically affecting its applicability, even when the object-oriented mapping algorithm is abstracted from defining a specific virtual object shape (Salvietti et al., 2014). In an interesting more recent mapping approach denoted as DexPilot (Handa et al., 2020), PH fingertip poses are obtained via a marker-less vision, and, thereafter, a cost function based on the distance and orientation among fingertips in the TH workspace is designed in order to optimize the final TH finger configurations. Other types of mapping are based on recognizing the PH posture in order to reproduce it on the TH (Ekvall and Kragic, 2004; Pedro et al., 2012; Ficuciello et al., 2021). In this kind of approach, the functions of the TH are limited to a discrete set of predefined grasps/motions and, thereafter, based on the output of the PH posture recognition process (mostly based on machine learning techniques (Dillmann et al., 2000; Yoshimura and Ozawa, 2012)), one of the available TH motions is selected and executed. In this case, the operator can easily learn how to configure ones own hand in order to activate specific actions of the robot hand, whereas the lack of continuous control, and the fact that the number of predefined TH grasps/motions increases with the complexity of the applications, *de facto* make this mapping method difficult to be applied for several applications (e.g., precision grasps and gestures/grasps not included in the predefined TH motions.) Furthermore, types of PH-to-TH mapping approaches can be found in the literature, for example, based on dimensionality reduction of the TH input space (Ciocarlie et al., 2007; Salvietti, 2018), deep learning architectures (Gomez-Donoso et al., 2019; Li et al., 2019), or shared control (Song et al., 1999; Hu et al., 2005). However, in general, state-of-the-art mapping solutions do not take into account the necessity of preserving on the TH, within a single method, both PH finger shapes and fingertip Cartesian positions. Instead, these aspects are essential to ensure acceptable levels of intuitiveness and predictability of TH motions during a hand motion mapping (Fischer et al., 1998), and we therefore claim that they should be carefully considered for novel advances in the research problem of mapping motions on robot hands.

In the light of these concepts, in this study, we introduce a hybrid approach combining joint and Cartesian mappings in a single solution. Specifically, the proposed hybrid joint–Cartesian mapping exploits specific spatial information that we denote as “available *in-hand*,” which means that is available *a priori* just once certain hand kinematics is given because they can be systematically derived from geometrical considerations and forward/inverse kinematics computations. We therefore exploit spatial in-hand information to enforce a smooth and continuous transition between joint and Cartesian mappings. In particular, in this study, we present and evaluate two specific realizations of this mapping paradigm. The first one is denoted as distance-based hybrid mapping, in which the spatial in-hand information related to the distance between the fingertips of the thumb and opposite fingers is exploited to realize the transition between joint and Cartesian mappings. In the second realization, denoted as workspace-based hybrid mapping, the spatial in-hand information used to enforce the transition between joint and Cartesian mappings is related to the areas of the workspace in which the thumb and opposite fingertips can get in contact. In the article, these hybrid mapping realizations are thoroughly presented, and simulation experiments are conducted on a series of well-known multi-articulated robot hands, both anthropomorphic and non-anthropomorphic, in order to evaluate and compare the proposed mapping paradigm.

## 2 Methods

### 2.1 Hybrid Joint–Cartesian Mapping Approach

**FIGURE 1:**
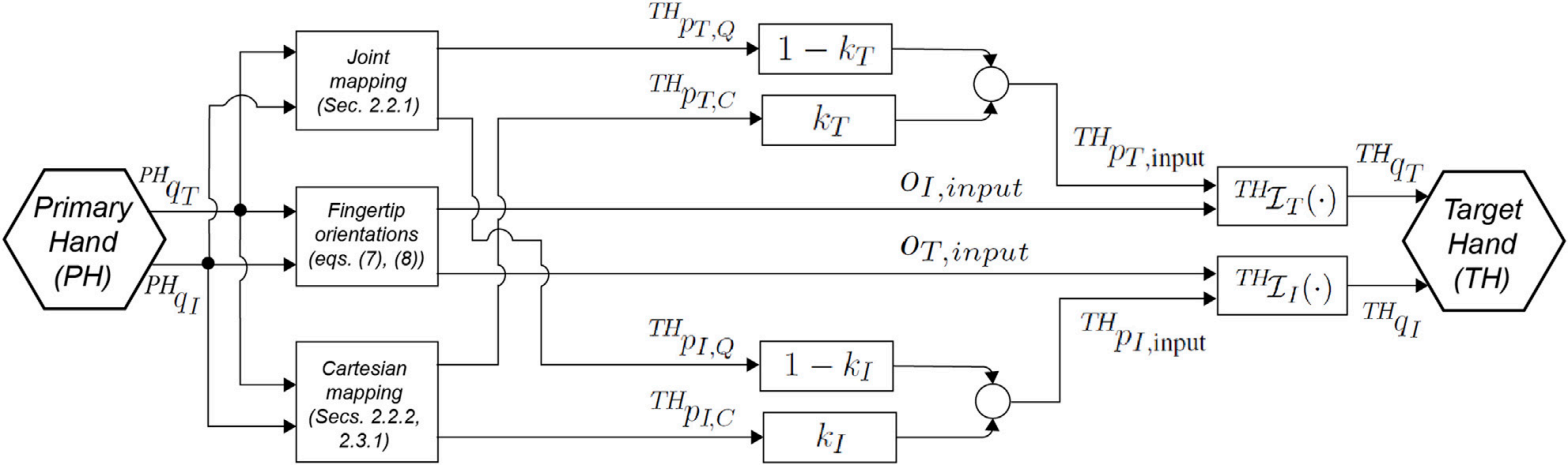
Block diagram of the proposed hybrid joint–Cartesian mapping approach, for thumb (subscript *i* = *T*) and index (subscript *i* = *I*) fingers only (extension to other fingers is straightforward.) The PH joints qiPH are mapped through joint and Cartesian mappings into TH fingertip coordinates pi,QTH and pi,CTH , respectively. A switching factor *k*_*i*_ allows the transition between joint and Cartesian mappings. TH finger’s inverse kinematics function Ii(⋅)TH allows to define the TH joint references qiTH from the finger position piTH and orientation *O*_*i*,*input*_.

We are interested in having a mapping algorithm to realize a smooth transition based on in-hand spatial information between two types of basic mappings, the joint mapping and the Cartesian mapping. Also, the transition has to be continuous in order to avoid any discontinuity during the PH-to-TH motion mapping. Therefore, we here introduce the general concept underlying the joint–Cartesian hybrid mapping based on spatial in-hand information and, thereafter, we will describe two specific realizations, the distance-based hybrid mapping and the workspace-based hybrid mapping, which characterize the usage of two different kinds of spatial in-hand information. To this aim, let us consider the block diagram of Figure 1, illustrating the general joint–Cartesian hybrid mapping approach. According to Figure 1, we will consider, here and in the following of this section, only the thumb and the index finger, since the algorithm can immediately be extended to the consideration of all PH and TH fingers. Furthermore, we assume in this section that the joint and Cartesian mappings results are already available, while their specific formulations for the distance-based realization and workspace-based realization are reported later in Section 2.2 and Section 2.3, respectively. Let us then consider qT,QTH and qI,QTH as the TH thumb and index joint values (right subscripts *T* and *I*, respectively), directly available from the PH (left superscript *T H*) as the application of the joint mapping (right subscript *Q*.) It is therefore possible to apply the TH forward kinematics of the thumb and index finger, denoted as FT(⋅)TH and FI(⋅)TH , respectively, to compute the Cartesian positions pT,QTH and pI,QTH and orientations oT,QTH and oI,QTH of the thumb and index fingertips, which result from the application of the PH-to-TH joint mapping (see Section 2.2.1). Note that oT,QTH and oI,QTH represent any suitable description of the fingertip orientations, for example, Euler angles or unit quaternions. It is therefore possible to compactly write the Cartesian positions and orientations as the fingertip pose vectors as follows:

xT,QTH=pT,QTHoT,QTHandxI,QTH=pI,QTHoI,QTH, (1)

for the TH thumb and index fingertips, respectively. Therefore, applying the forward kinematics it holds that xT,QTH=FTTH(qTHT,Q) and xI,QTH=FITH(qI,QTH) .

In a similar manner, if instead of the joint mapping we consider the case of the Cartesian mapping, we can write the TH thumb and index fingertip poses as:

xT,CTH=pT,CTHoT,CTHandxI,CTH=pI,CTHoI,CTH, (2)

respectively, where the fingertip Cartesian positions pT,CTH,pI,CTH and orientations oT,CTH,oI,CTH are available from the application of the PH-to-TH Cartesian mapping (see Section 2.2.2 and Section 2.3.1) Now, considering qTTH and qITH the actual thumb and index joint values, respectively, we can write them as imposed by their inverse kinematics functions IT(⋅)TH and II(⋅)TH , that is,

qTTH=ITTHxT,inputTH, (3)

qITH=IITHxI,inputTH, (4)

where

xT,inputTH=pT,inputTHoT,inputTHandxI,inputTH=pI,inputTHoI,inputTH, (5)

with pT,inputTH,pI,inputTH and oT,inputTH,oI,inputTH being the Cartesian position and orientation inputs for the thumb and index finger, respectively. Therefore, in order to implement a smooth transition between joint and Cartesian mappings, in our mapping algorithm we impose that

pT,inputTH=1−kTpT,QTH+kTpT,C,THpI,inputTH=1−kIpI,QTH+kIpI,C.TH (6)

In Eq. 6, *k*_*T*_ and *k*_*I*_ are smooth, sigmoidal gains governing the transition between joint and Cartesian mappings. Since we want our hybrid mapping to be based on spatial information, it follows that the definition of *k*_*T*_, *k*_*I*_ has to be founded on such spatial information, and therefore they will be introduced in the next subsection, with different formulations for the distance-based (see Section 2.2.3) and workspace-based (see Section 2.3.2) hybrid mapping realizations. Now that we have introduced how the Cartesian position inputs for the TH thumb and index fingertips are imposed by the proposed mapping algorithm, we are going to describe the fingertips' orientation inputs (see also Figure 1) we imposed.

oT,inputTH=oT,QTH,ifkTI=0oT,map,THotherwise, (7)

oI,inputTH=oI,QTH,ifkTI=0oI,map,THotherwise, (8)

where oT,mapTH and oI,mapTH are the orientations of the PH thumb and index fingertips with respect to their finger base reference frames, that, if necessary, has to be consistently described with respect to the TH index and thumb base reference frames by using proper homogeneous transformation matrices (Siciliano et al., 2010). In this relation, we assume that an iterative, gradient-based inverse kinematics algorithm is used to prioritize the fingertip position with respect to the orientation targets, in such a way that an admissible orientation (with smallest joint deviation) is obtained in case that the imposition of a certain fingertip orientation on the TH is incompatible with the imposition of the fingertip position (Sugihara, 2009). Note that, when presenting, in the following, the specific implementation of the Cartesian mappings for the distance-based and workspace-based mapping realizations, we will refer only to the fingertip position, since the fingertip orientation is imposed according to Eqs. 7, 8.

### 2.2 Distance-Based Hybrid Mapping Realization

In the distance-based realization of the proposed hybrid mapping, we consider the spatial in-hand information given by the module of the distance vector between the thumb and opposite fingertips. The idea is that, during a wide variety of precise actions, the thumb fingertip is closer in space to opposite fingertips. Thus, when the distance between the PH thumb and index fingertips (the algorithm can be immediately extended to any thumb-finger couple) is lower than a given threshold, a transition between joint and Cartesian mappings is imposed on the TH. In the following joint mapping, Cartesian mapping and transition behavior are illustrated.

#### 2.2.1 Joint Mapping

Let us denote with qTPH,qIPH and qTTH,qITH the vectors of joint values for the PH and SH thumb and index fingers, respectively. We define the PH-to-TH joint mapping as:

qT,iTH=qT,h,PHwithi=1,…,m∧∀i,h∈1,…,n, (9)

qI,jTH=qI,kPH,withj=1,…,l∧∀j,k∈1,…,p, (10)

Where *m*, *n*, *l*, and *p* are TH thumb, TH index, PH thumb, and PH index number of joints, respectively. In other words, Eqs. 9, 10 describe that, to each single joint of the TH thumb and index fingers (denoted by the subscripts *i* and *j* in Eqs. 9, 10) is arbitrarily imposed the angular value related to a single specific joint of the PH thumb or index fingers (denoted by the subscripts *h* and *k* in Eqs. 9, 10). According to Eq. 10, qTTH and qITH are therefore available, according to the joint mapping of the distance-based mapping realization, for being used in the algorithm schematized in Figure 1 and described in Section 2.1.

#### 2.2.2 Cartesian Mapping

Within the distance-based hybrid mapping realization, the Cartesian mapping is implemented considering differently the thumb and the opposite fingers. Taking into account the index finger, as representative of the opposite fingers without loss of generality, its Cartesian mapping is realized according to the following relation:

pI,CTH=cspIPH−pTPH+pT,CTH, (11)

where pTPH and pIPH are the PH thumb and index fingertip positions, respectively, *c*_*s*_ is a scaling constant set as the ratio between TH and PH thumb lengths, and pT,CTH is given according to the TH thumb Cartesian mapping as reported in the following. Taking into account the *x*, *y*, and *z* components of the PH and TH thumb and index fingertip positions, we can consider that pvPH=[pvxPHpvyPHpvzPH]T and pvTH=[pvxTHpvyTHpvzTH]T , with *v* = {*T*, *I*} indicating the thumb and index fingers. Then, we define

dl,t=‖pOtxTH−pOlxTH‖‖pOtxPH−pOlxPH‖ (12)

and the PH thumb position relative to the base frame of the opposite finger *m* is:

pT,mPH=pTPH−pOmPH, (13)

where the subscripts *l*, *t*, and *m* are equal to {*l*, *t*} = {{*I*, *M*}, {*M*, *R*}, {*R*, *P*}}, and *m* = {*I*, *M*, *R*}, with *I*, *M*, *R*, and *P* indicating the index, middle, ring, and little fingers, and pOmPH,pOmTH indicate the PH and TH positions of the origin of the base frame of the finger *m*. Then, the Cartesian mapping for the TH thumb is imposed as

pT,CxTH=TOITHcspT,IPHx,ifpTxPH≥pOIx,PHTOITHpT,IPHx+dI,MpT,IPHx,ifpOMxPH<pTxPH≤pOIx,PHTOMTHpT,MPHx+dM,RpT,MPHpT,Mx,ifpORxPH<pTxPH≤pOMx,PH[TORTHpT,RPH]x+d{R,P}[pT,RPH]x,ifpOPxPH<pTxPH≤pORx,PHTOPTHcspT,PPHx,ifpTxPH<pOPx,PH,, (14)

pT,CyTH=TOITHpT,IPHcsy, (15)

pT,CzTH=TOITHpT,IPHcsz, (16)

**FIGURE 2:**
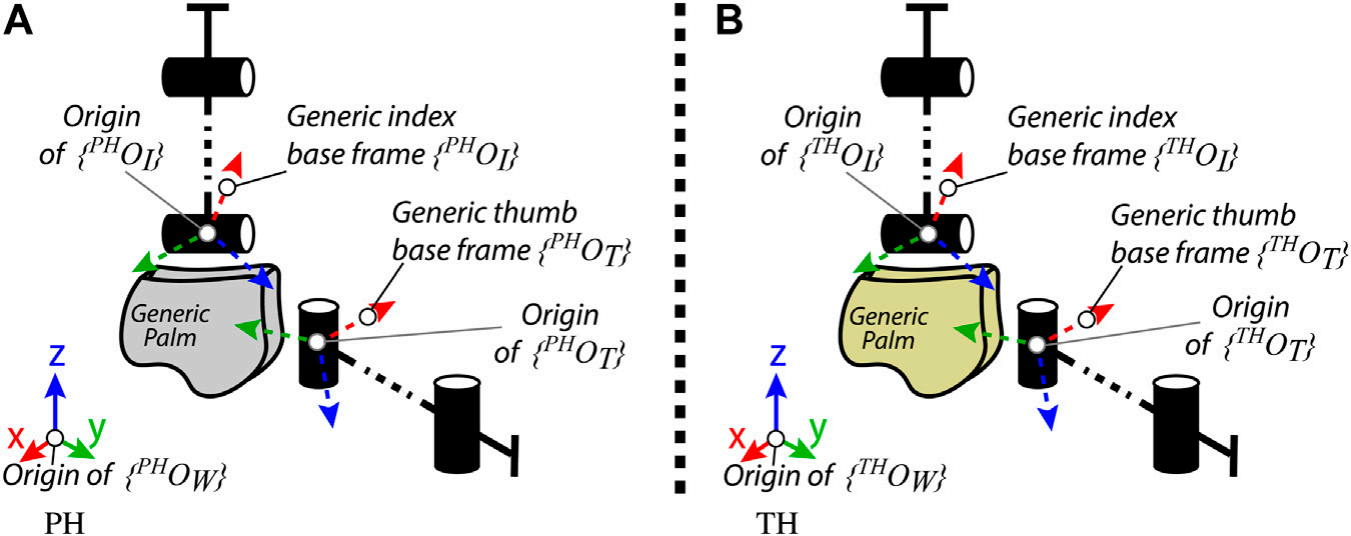
Generic representations of the **(A)** PH and **(B)** TH thumb and index finger, for the distance-based hybrid mapping realization.

Where the [*a*]_*b*_ indicates the component along the *b* axis of the vectorial expression *a*, TOwTH , with *w* = {*I*, *M*, *R*, *P*} denoting the homogeneous transformation describing the TH *w* finger’s base frame {OwTH} with respect to the TH world frame. In other words, according to Eqs. 14 and 15 and 16, the Cartesian mapping of the TH thumb is realized such that to preserve the distance between the PH thumb fingertip and the origin of the base frames of the opposite fingers, along the *x* component. Indeed, the value of the *x* component of the position of the TH thumb fingertip is imposed according to five different condition in Eq. 14, which, from top to bottom, have the following meaning: 1) the thumb fingertip is located at the right of the index finger base frame origin; 2) the thumb fingertip is located between the index and middle finger base frame origins; 3) the thumb fingertip is located between the middle and ring finger base frame origins; 4) the thumb fingertip is located between the ring and pinkie finger base frame origins; and 5) the thumb fingertip is located at the left of the pinkie finger base frame origin. Instead, the Cartesian mapping of the TH thumb *y* and *z* components is chosen based on the distance between the PH thumb fingertip and the base frame origins of the PH index finger (see Figure 2.)

#### 2.2.3 Transition

In order to define the joint–Cartesian mapping transition of the distance-based hybrid mapping realization, it is necessary to properly impose the gains *k*_*T*_ and *k*_*I*_ previously introduced in Eq. 6 in Section 2.1 (see also Figure 1.) To this purpose, let *r*_in_ and *r*_out_ (*r*_in_ < *r*_out_) be the radii of two spheres centered in the PH thumb fingertip. For the TH index finger, we want to obtain that: *1)* the joint mapping (see Section 2.2.1) is applied if the inequality ‖pTPH−pIPH‖>rout is satisfied and 2*)* the Cartesian mapping (see Section 2.2.2) is applied if the condition ‖pTPH−pIPH‖<rin is matched, and 3) a transition between joint and Cartesian mappings is realized if rin≤‖pTPH−pIPH‖≤rout . Therefore, the gains are enforced as:

$$k_{T}=1,$$ (17)

$$k_{I}=\left\{\begin{array}{cc} 1\; & \text{if}\;\delta_{I}<r_{\text{in,}}\\ \frac{1}{2}\left(1-\cos\left(\frac{\delta_{I}\pi }{r_{\text{out}}-r_{\text{in}}}\right)\right)\; & \text{if}\;r_{\text{in}}\leq \delta_{I}\leq r_{\text{out,}}\\ 0\; & \text{if}\;\delta_{I}>r_{\text{out}} \end{array}\right.,$$ (18)

with δI=‖pTPH−pIPH‖ , where *k*_*T*_ is constantly set to 1 because for the TH thumb finger, the Cartesian mapping is always applied, and *k*_*I*_ presents a sigmoid-like profile for *r*_in_ ≤ *δ*_*I*_ ≤ *r*_out_.

### 2.3 Workspace-Based Hybrid Mapping Realization

In the workspace-based realization, the spatial in-hand information exploited for the implementation of the joint–Cartesian hybrid mapping is related to the areas of the PH and TH workspaces in which the thumb and index can get in contact (as before, we keep considering only the thumb and index finger without the loss of generality). Indeed, the consideration of the fingertip contact areas within robot hands has not been investigated in previous mapping methods, while the intersection between finger workspaces has been demonstrated to be a key descriptor in dexterous manipulations (Kuo et al., 2009), and therefore we want to explore its usage as spatial information within the proposed hybrid mapping approach. In the following description of the workspace-based mapping realization, the joint mapping is not illustrated because its formulation has no differences from the joint mapping already described in Section 2.2.1 for the distance-based mapping realization. Therefore, in the following, only the specific implementation of the Cartesian mapping and joint–Cartesian mapping transition is provided.

#### 2.3.1 Cartesian Mapping

**FIGURE 3:**
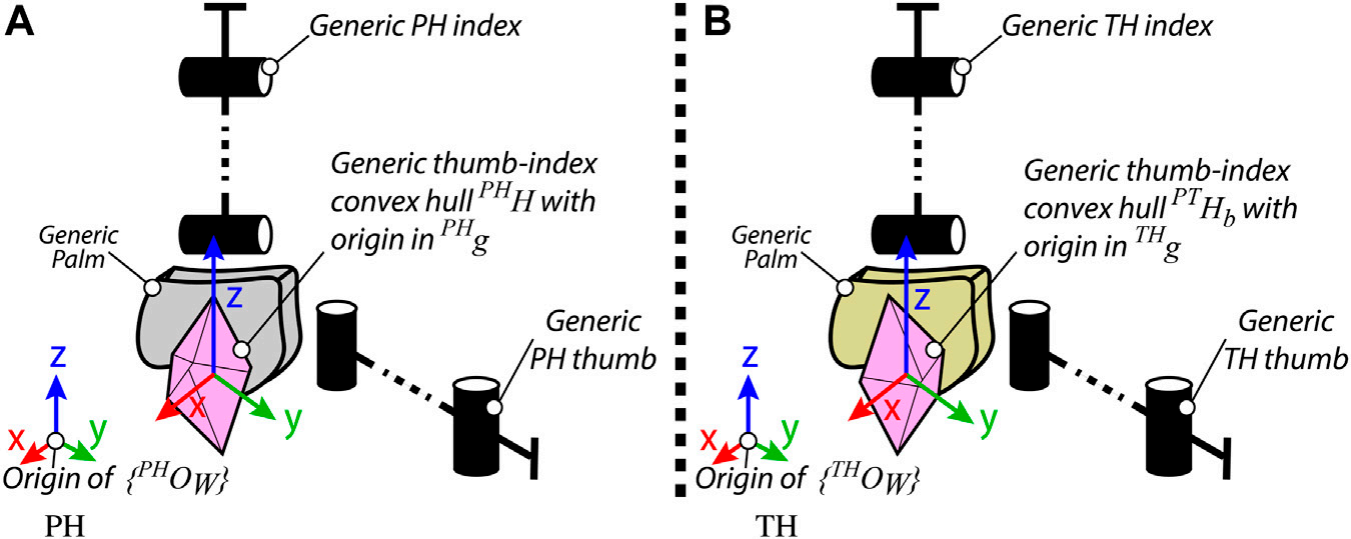
Generic representations of the **(A)** PH and **(B)** TH thumb and index finger, for the workspace-based hybrid mapping realization.

Let us consider the PH workspace region delimited by the convex hull HPH , corresponding to the set of discretized workspace points denoted by BHPH⊇HPH , and obtained as the intersection of the discretizations of the PH thumb and index workspaces. We refer to gPH as the centroid of HPH . Note that, by default, we consider that the introduced geometric entities are implicitly described with respect to a PH world reference frame {OWPH} . Then, let us define the convex hull H∗PH and its centroid g∗PH , as the representation of HPH with respect to a different reference frame {GPH} , placed in gPH and with the *y*–*z* and *x*–*z* planes parallel to the PH index’s frontal plane and sagittal median plane, respectively (see Figure 3.) We then denote with TGPH the homogeneous transformation matrix describing {GPH} with respect to {OWPH} . Considering now the TH, it is possible to define, in the same manner as for the PH, the convex hull HTH and its centroid gTH . In this relation, we are interested in placing a reference frame {GTH} in gTH , with its *y*–*z* and *x*–*z* planes parallel to the TH index’s frontal plane and sagittal median plane, respectively (see Figure 3), such that to able to consider the convex hull H∗TH as the description of HTH with respect to {GTH} . The description of {GTH} with respect to the TH world frame {GTH} is therefore given by the homogeneous transformation matrix TGTH . It follows that can we can define a convex hull H∗PT , obtained by placing the points of H∗PH within the TH workspace as described with respect to {GTH} and then denote the set HPT as the description of H∗PT with respect to the TH world frame {GTH} . Note that, in other words, the latter convex hull {GTH} is nothing but the PH convex hull relocated in gTH . Additionally, we consider HbPT as the convex hull HPT scaled by a factor *b* and its description Hb∗PT with respect to {GTH} . In particular, the scaling factor *b* is chosen such that the extension of H∗TH along the *x* axis is contained within the area delimited by Hb*PT (see Figure 3.) Then, in order to describe the Cartesian mapping and according to the concepts introduced so far, we denote by pTPT and pIPT the fingertip positions of the PH thumb and index finger as resulting from the following steps applied in sequence: 1*)* they are described with respect to {GPH} , 2) then imposed with respect to the frame {GTH} in the TH workspace, 3) and finally described with respect to {OWTH} . In formal terms, these steps are described by the following expressions:

pTPT=TGTHTGPH−1pTPH,pIPT=TGTHTGPH−1pIPH. (19)

The Cartesian mapping is finally imposed for the TH thumb and index finger as

pTTH=bpTPT−gTH+gTH,pITH=bpIPT−gTH+gTH. (20)

#### 2.3.2 Transition

For the description of the joint–Cartesian mapping transition for the workspace-based realization of the proposed hybrid mapping, let us define HsPH as the convex hull HPH scaled by a factor *s* > 1, and let BHsPH be the set of discretized points of the workspace portion delimited by HsPH . It is then possible to consider the set BH=BHsPH\BHPH , which contains the portion of BHsPH that does belong to BHPH . Therefore, a function $f:B_{H}\subset R^{3}\to R$ performing a multivariate interpolation between the points of HPH and HsPH can be defined, such that 1*)* f(h)=0∀h∈HsPH , 2*)* f(h)=1∀h∈HPH , 3*)* and linearly interpolated values of f(h),∀h∈BH\HsPH . Specifically, we compute the function *f* by solving the described multivariate interpolation problem as the radial basis function (RBF) interpolation problem (Chen et al., 2005). Exploiting the interpolation function *f*, we define another function, the transition function fTI(pTPH,pTPH) , as:

fTIpTPH,pTPH=fpTPH,iffpTPH≤fpIPHfpIPH,iffpTPH>fpIPH, (21)

which can also be simply indicated as *f*_*TI*_. We can now finally describe the gains *k*_*T*_ and *k*_*I*_, as necessary to impose a desired joint–Cartesian transition (see Eq. 6 in Section 2.1 and Figure 1), which are given according to

kT=kI=0,ifpTPH,pIPH∉BHsPH1,ifpTPH,pIPH∈BHPH121−cosπfTI,ifpTPH,pIPH∈BH. (22)

According to Eq. 22, the Cartesian mapping is enforced if pTPH and pIPH are inside the workspace area delimited by the convex hull HPH , whereas the joint mapping is applied if pTPH and pIPH are outside of the workspace region delimited by HsPH , and a joint–Cartesian transition takes place, according to Eqs. 6, 22, when both pTPH and pIPH are in the workspace portion between HPH and HsPH .

## 3 Experiment and Results

**FIGURE 4:**
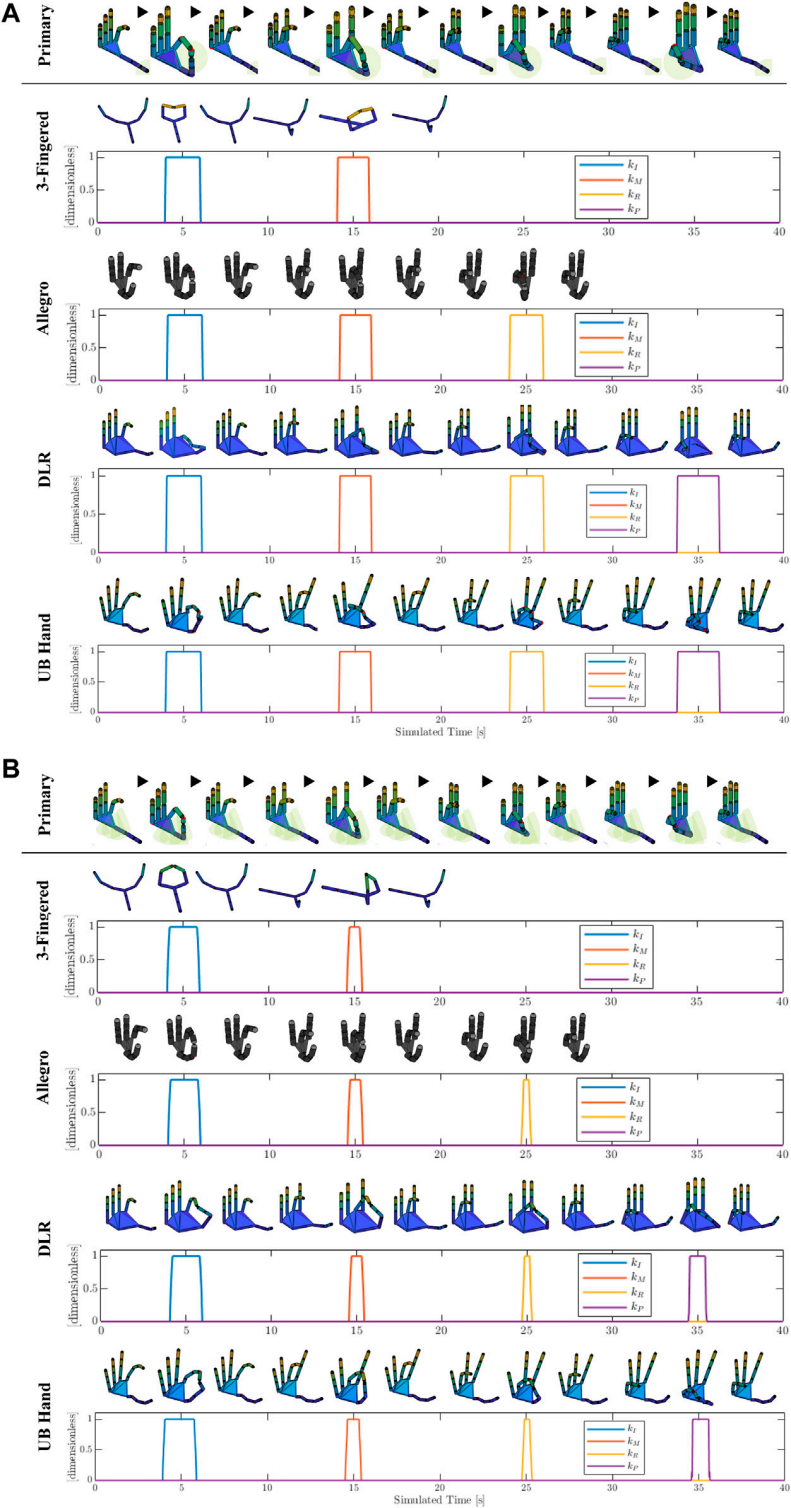
Mapping of the tip-to-tip motion of the PH on the different types of TH and related transition gains between joint and Cartesian mappings (*k*_*I*_, *k*_*M*_, *k*_*R*_, and *k*_*P*_ for the index, middle, ring, and pinkie fingers, respectively) for the **(A)** distance-based and **(B)** workspace-based proposed approaches. The sphere of the distance-based mapping and the convex hull of the workspace-based mapping (see Section 2) are reported only on the PH for better visualization. Note that a color-coded shading of the simulated robot hand is applied for improving the visualization.

For the evaluation of the proposed PH-to-TH mapping strategies, we performed a series of simulation experiments using both non-anthropomorphic and anthropomorphic robot hands. As PH, we used the paradigmatic hand model, available from the open-source SynGrasp MATLAB toolbox (Malvezzi et al., 2015) (see Figure 4.) As TH, we simulated, via SynGrasp, four types of well-known multi-articulated robot hands: a 3-fingered hand inspired to the Barrett hand (Townsend, 2000), the 4-fingered anthropomorphic Allegro hand (WonikRobotics, 2015), the anthropomorphic DLR-Hit II hand (Butterfaß et al., 2001), and the anthropomorphic University of Bologna Hand IV (UBHand) (Melchiorri et al., 2013) (Figure 4).

Specifically, for the first simulation experiment, a particular motion was generated for the PH, referred to as tip-to-tip motion, in which the fingertips of the thumb and opposite fingers get in contact in a sequence, as shown in Figure 4. Furthermore, Figure 4 also reports the results of the distance-based (Figure 4A) and workspace-based mappings (Figure 4B) on the different simulated robot hands, along with the relative behavior of the gains for the transition between joint and Cartesian mappings (*k*_*I*_, *k*_*M*_, *k*_*R*_, and *k*_*P*_ for the index, middle, ring, and pinkie fingers, respectively.) Note that for the 3-fingered hand we mapped only the PH thumb, index, and middle fingers, whereas for the Allegro hand we mapped only the PH thumb, index, middle, and ring fingers. From Figure 4, first of all, it is possible to see how both the distance-based and workspace-based mappings allow the TH to conserve the shape of the PH fingers (i.e., their joint configuration) when the fingers are not close to the thumb, and, at the same time, to perform a correct thumb–fingers tip-to-tip contact, thanks to the switching to the Cartesian mapping according to the related transition gains.

In Figure 6A we reported, for each different TH, the mean joint error—that is the mean of the norm of the error between the joint angle vectors of the PH and the TH—during the enforcing of the Cartesian mapping (top graph, for both distance-based and workspace-based approaches), and the mean thumb–finger fingertip Cartesian position error during the enforcing of the joint mapping (bottom graph, for both distance-based and workspace-based approaches). Note that the latter Cartesian error was normalized by the value of the distance between thumb and index fingertips in the open hand configuration, for each respective robot hand. This made the Cartesian error between the different robot hands comparable, taking into account the different TH sizes. Looking at the top graph of Figure 6A, it is possible to observe that for the workspace-based mapping the Allegro hand presented a clearly higher joint error when the workspace-based mapping was used, due to the fact that the Allegro hand presents a workspace geometrically less compatible with the one of the PHs (the paradigmatic hand.) Also, it is possible to see how 5-fingered anthropomorphic robot hands showed a lower joint error. Looking at the bottom graph of Figure 6A, it is worth highlighting that, using the workspace-based approach, the 3-fingered hand and the Allegro hand showed a substantially higher thumb–finger Cartesian error. This was due to the fact that, since these two TH present more pronounced kinematic differences with respect to the PH anthropomorphic structure and therefore more different workspaces, the distance-based mapping enforces the Cartesian mapping for a larger part of the motion, reducing the reported Cartesian error (which is reported when the joint mapping is enforced). Differently, the workspace-based approach required that both the thumb and opposite finger enter the convex hull region which enables the transition to the Cartesian mapping.

**FIGURE 5:**
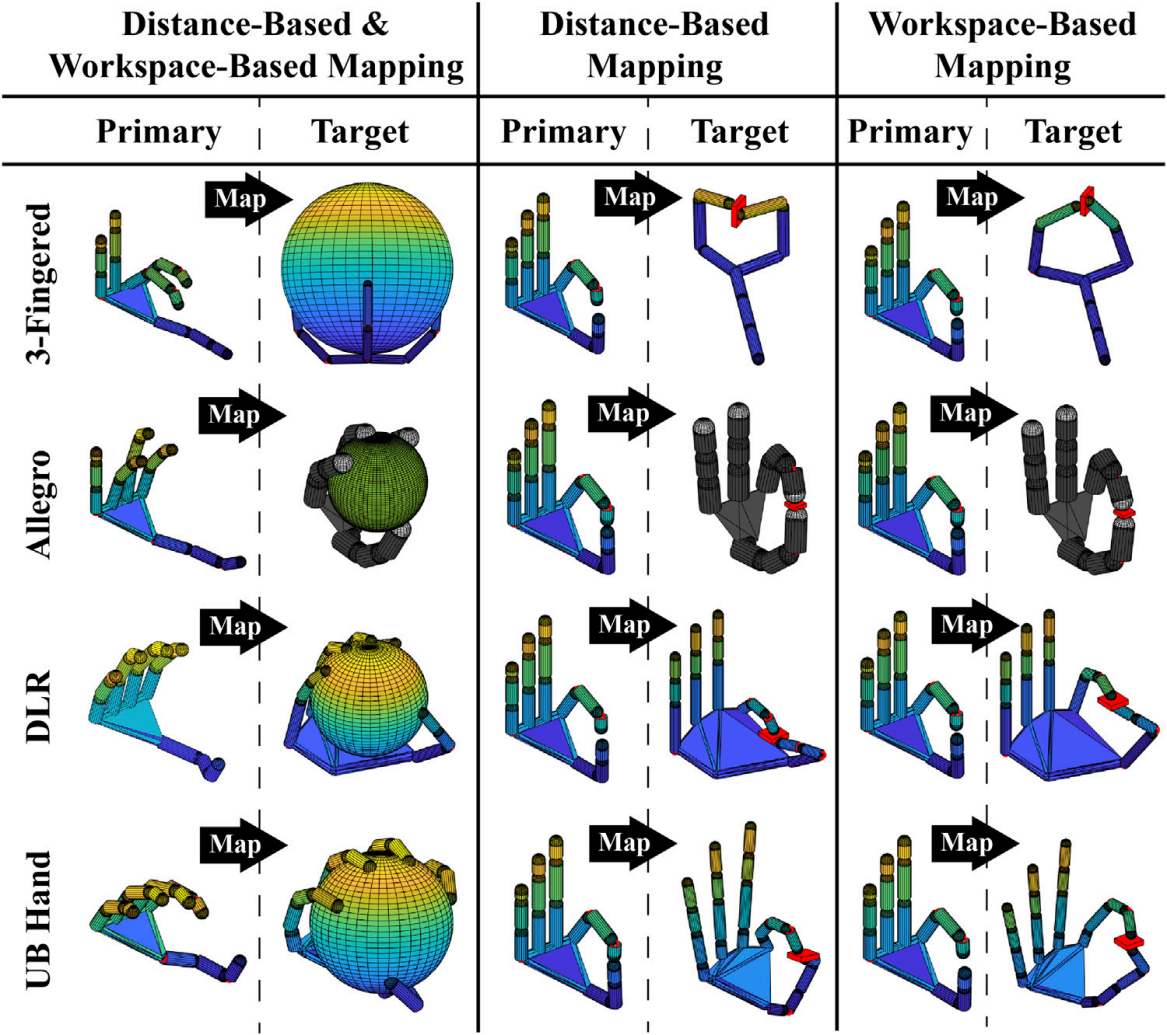
Proposed mapping approaches applied for the simulated grasp of a sphere (left part) and a thin cylinder (right part). Note that a color-coded shading of the simulated robot hand is applied for improving the visualization.

**FIGURE 6:**
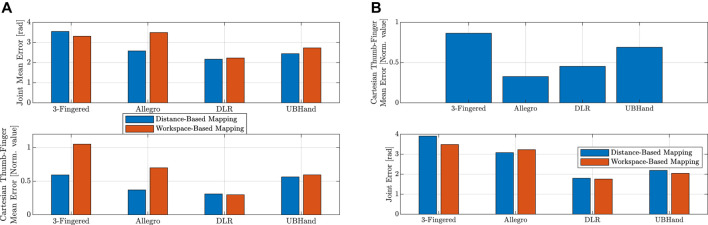
**(A)** Top graph: mean joint error for the tip-to-tip motion during the enforcing of the Cartesian mapping. **(A)** Bottom graph: mean Cartesian error for the tip-to-tip motion during the enforcing of the joint mapping. **(B)** Top graph: Cartesian error for the grasping of the spherical objects. **(B)** Bottom graph: joint error for the grasping of the thin cylinder object.

The second simulation experiment, visible in Figure 5, consisted in performing the grasp of a sphere requiring to use the joint mapping for the preservation of the PH finger shapes and a thin cylinder requiring precise positioning of the fingertip in order to preserve the fingertips–object contact point locations. For the grasping of the sphere, the PH fingers were closed uniformly along all joints producing the finger flexions. Then, the motion was mapped on the different simulated robot hands (distance-based and workspace-based mappings coincide, in this case, because only joint mapping was enforced in both mappings), and each PH finger was stopped when the relative TH finger got in contact with the object surface. The result of the sphere grasping can be seen in the left part of Figure 5 for the different robot hands, and the Cartesian error is reported in the top graph of Figure 6B. In the latter graph, it can be observed how the 3-fingered hand showed the higher Cartesian error, due to the fact that it presents the less anthropomorphic-like kinematic structure. For the grasping of the thin cylinder, the tip-to-tip motion only for the thumb and index finger was performed on the PH and then mapped with the distance-based and workspace-based approaches on the TH. The PH thumb and index were stopped when the TH counterpart got in contact with the object's surface. As can be seen in the right part of Figure 5, both proposed mapping methods were able to map the precision grasp on the different robot hands (the position of the cylinder was adjusted based on the specific TH because we reasonably assumed that, during the thumb–index approaching motion, the object location could be adjusted with respect to the robot hand accordingly). The bottom graph of Figure 6B reports the joint error when the cylinder is grasped. It is possible to see that, for the different robot hands, the distance-based and workspace-based mappings showed comparable joint errors, with the workspace-based mapping presenting lower error values for the 3-fingered, DLR, and UBHand robot hands. This latter aspect can also be seen in the right part of Figure 5, where the workspace-based approach was able to put the TH fingers in contact with the object with a more similar shape with respect to the PH. A video of the simulation results reported in Figures 4, 5 is available at the link: https://bit.ly/3OUcqaa.

Finally, in order to contextualize the proposed hybrid mapping approaches with respect to other mapping methods presented in the literature, we report a qualitative comparison. Specifically, in Table 1, we report a comparison between the proposed mapping, standard joint/Cartesian mappings, and two recent advanced mappings. The considered mappings are the following: a standard direct joint mapping in accordance with Speeter (1992), a standard direct Cartesian mapping as presented by Rohling and Hollerbach, (1993), a virtual-object-based mapping Gioioso et al. (2013), and the DexPilot mapping (Handa et al., 2020). Specifically, the qualitative comparison was made with respect to the following features: 1) PH-to-TH preservation of the finger shapes, 2) PH-to-TH preservation of the distance between thumb and opposite fingertips, 3) applicability to anthropomorphic TH, 4) applicability to non-anthropomorphic TH, 5) evaluation on multiple hands (with different kinematic structures and number of fingers), and 6) evaluation metrics (see Table 1). Looking at the table, it can be observed how only the proposed mappings and the DexPilot mapping (Handa et al., 2020) have the capability to preserve both PH finger shapes and fingertip positions on the TH. In our approaches, different from the others, in Table 1, we also evaluated these capabilities on different robot hand kinematic structures, both anthropomorphic and non-anthropomorphic, by means of the simulation study presented in this section.

**TABLE 1:** Qualitative comparison between the proposed mapping and other representative mappings.

	Proposed hybrid mapping realization	Direct joint mapping (Speeter, 1992)	Direct Cartesian mapping (Rohling and Hollerbach, 1993)	Virtual object mapping (Gioioso et al., 2013)	DexPilot mapping (Handa et al., 2020)
**PH-to-TH preservation of the finger shapes**	During volar/power grasps and gestures	Yes	No	No	Yes
**PH-to-TH preservation of the distance between thumb and opposite fingertips**	During precision grasps and gestures	No	Yes	Yes	Yes
**Applicability to anthropomorphic TH**	Yes	Yes	Yes	Yes	Yes
**Applicability to non-anthropomorphic TH**	Yes	Yes	Yes	Yes	Yes
**Evaluation of multiple TH**(**with different structures and number of fingers**)	Yes simulation study	No	No	Yes simulation study	No
**Evaluation metrics**	Joint and Cartesian errors during simulated motions and grasps	N.A.	Success rate of telemanipulation tasks	Contact forces of simulated grasps	Completion time and success rate of telemanipulation tasks

## 4 Conclusion

Two realizations of a joint–Cartesian hybrid mapping for robot hands, based on spatial in-hand information, have been presented in this article: the distance-based and workspace-based approaches. By performing several simulation experiments of PH-to-TH mapping, we evaluated the proposed approaches on four well-known robot hands: a 3-fingered hand inspired by Barrett hand, the 4-fingered anthropomorphic Allegro hand, the anthropomorphic DLR-Hit II hand, and the anthropomorphic UBHand. We evaluated both a free motion of the PH including thumb–fingers tip-to-tip contacts and the grasping of a sphere (requiring finger shape preservation) and a thin cylinder (requiring fingertip position preservation.) The results show that both approaches were capable of successfully performing the mapping of PH finger shapes and TH fingertip positions, on both anthropomorphic and non-anthropomorphic robot hands, and the differences between the distance-based and workspace-based approaches have been discussed. Future developments will be devoted to performing experiments with real robot hands, focusing the study on the investigation of specific telemanipulation performance using the proposed mapping, instead of a simulative evaluation of multiple different robot hands.

## Data Availability

The raw data supporting the conclusion of this article will be made available by the authors, without undue reservation.
